# Influence of Amino Acid Substitutions in Capsid Proteins of Coxsackievirus B5 on Free Chlorine and Thermal Inactivation

**DOI:** 10.1021/acs.est.3c10409

**Published:** 2024-03-15

**Authors:** Shotaro Torii, Jérôme Gouttenoire, Kiruthika Kumar, Aleksandar Antanasijevic, Tamar Kohn

**Affiliations:** † Laboratory of Environmental Chemistry, School of Architecture, Civil and Environmental Engineering (ENAC), École Polytechnique Fédérale de Lausanne (EPFL), CH-1015 Lausanne, Switzerland; ‡ Division of Gastroenterology and Hepatology, Lausanne University Hospital and University of Lausanne, CH-1011 Lausanne, Switzerland; § Virology and Structural Immunology Laboratory, School of Life Sciences, École Polytechnique Fédérale de Lausanne (EPFL), CH-1015 Lausanne, Switzerland

**Keywords:** coxsackievirus B5, disinfection, enterovirus, reverse genetics, water treatment

## Abstract

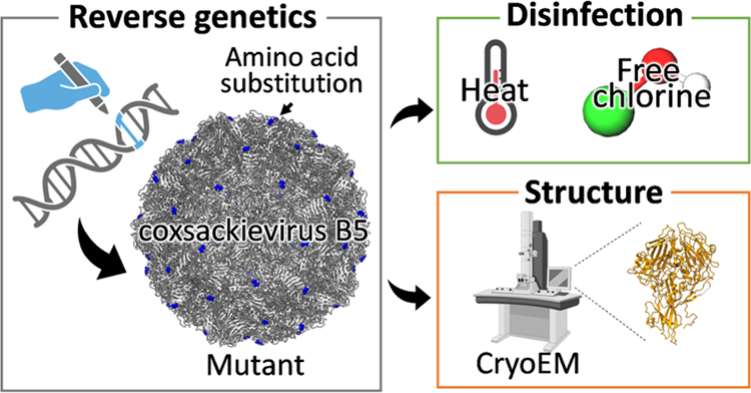

The sensitivity of enteroviruses to disinfectants varies among genetically similar variants and coincides with amino acid changes in capsid proteins, although the effect of individual substitutions remains unknown. Here, we employed reverse genetics to investigate how amino acid substitutions in coxsackievirus B5 (CVB5) capsid proteins affect the virus’ sensitivity to free chlorine and heat treatment. Of ten amino acid changes observed in CVB5 variants with free chlorine resistance, none significantly reduced the chlorine sensitivity, indicating a minor role of the capsid composition in chlorine sensitivity of CVB5. Conversely, a subset of these amino acid changes located at the C-terminal region of viral protein 1 led to reduced heat sensitivity. Cryo-electron microscopy revealed that these changes affect the assembly of intermediate viral states (altered and empty particles), suggesting that the mechanism for reduced heat sensitivity could be related to improved molecular packing of CVB5, resulting in greater stability or altered dynamics of virus uncoating during infection.

## Abstract 2

Amino acid substitutions in the capsid protein of CVB5 minimally affect chlorine resistance but notably reduced heat sensitivity, possibly due to enhanced capsid stability and/or altered infection dynamics.

## Introduction

Enteroviruses are nonenveloped positive single-stranded (ss) RNA viruses and are major causative agents of waterborne diseases. Coxsackievirus B5 (CVB5) is a genotype of the *Enterovirus* genus, which is frequently detected in clinical and wastewater surveillance.^1,2^ CVB5 is further divided into two lineages according to the phylogenetic similarities of the viral protein (VP) 1 gene:^3^ genogroup A, which includes the commercially available Faulkner strain, and genogroup B. Uniquely, CVB5 demonstrates a markedly lower sensitivity to common disinfectants, such as free chlorine and heat, compared to other waterborne viruses,^4−9^ making it a challenging virus to control in disinfection processes.

Most studies to date have focused on a single CVB5 variant, the Faulkner strain, to understand CVB5 inactivation kinetics^4,6,10−12^ and to investigate inactivation mechanism^13^ by disinfectants. It is increasingly apparent, however, that genetically diverse CVB5 environmental isolates differ in their sensitivity to chlorine and heat.^7,14^ Variant-dependent sensitivity to disinfectants has also been reported for other genotypes of enterovirus.^14,15^ Furthermore, variants also exhibit differing susceptibilities to other disinfectants,^16^ whereby the extent of the difference has been found to depend on the mechanism of action exerted by the disinfectant.^7,15,17^

Free chlorine has been found to oxidize the viral capsid,^18,19^ which protects the viral genome from chemical and enzymatic damage,^20,21^ and has been reported to inhibit the viral attachment function, thereby partially contributing to enterovirus inactivation.^13,17,22,23^ Sulfur-containing amino acids (cysteine (Cys) and methionine (Met)) react with free chlorine much faster than other amino acid residues.^24,25^ Thus, differing abundances and solvent-accessibilities of Cys and Met in capsid proteins have been suggested as a rationale for the varying virus sensitivities to commonly used oxidants.^7,15,19,26−30^ This theory is consistent with the findings on the free chlorine susceptibility of 13 environmental isolates of CVB5, where isolates belonging to genogroup B, which contain fewer Met in the capsid proteins, also exhibited a 1.9-fold lower sensitivity to free chlorine compared to isolates falling within genogroup A.^28^

In contrast to the chemical changes induced by free chlorine, the mechanism of thermal inactivation of enterovirus is based on structural modifications. Specifically, heat treatment can induce partial disassembly of enterovirus capsids into subunits or trigger a conformational rearrangement equivalent to viral uncoating (i.e., transition from the mature closed (F) state to the altered-intermediate (A) state),^31−34^ thereby resulting in virus inactivation. This latter mechanism is suggested to be the primary driver for thermal inactivation of CVB5 at 55 °C.^33^ Enhanced thermotolerance can be achieved by improving the stability of individual capsid building blocks (VP1–4) or strengthening the interaction network at the interfaces.^35^ Consequently, enhanced capsid protein interactions were proposed to rationalize differences in the thermotolerance of different enteroviruses genotypes.^33^ An alternative mechanism for thermotolerance was proposed for poliovirus 1 (PV1), where amino acid substitutions in the VP1 hydrophobic pocket region stabilized the capsid against inactivation by heat by limiting premature uncoating and release of viral RNA.^36^ An amino acid substitution in the pocket region, M180V at VP1, was also found after experimental evolution of CVB5 to attain thermotolerance.^37^

Despite the distinctly different inactivation mechanisms exerted by free chlorine and heat, the chlorine and heat resistances of enteroviruses have been found to coincide. Among the different enterovirus strains investigated in our past work,^7^ the least chlorine-sensitive genotypes (CVB1 and CVB5) were also the least heat-sensitive, and the most chlorine-sensitive genotype (echovirus 11; E11) was also the most heat-sensitive. Furthermore, a reduced sensitivity to free chlorine was also observed when E11 was experimentally adapted to greater heat tolerance.^38^ Finally, the amino acid change identified in heat-adapted CVB5 (M180V in VP1) was also observed in chlorine-resistant viruses.^28^ However, the viral features associated with reduced chlorine sensitivity, heat sensitivity, and the coincidence of the two remain unknown.

Identifying the role of individual amino acid substitutions in disinfection tolerance has been challenging, as resistant mutants typically exhibit several changes simultaneously. A powerful tool to overcome this challenge is the use of a reverse genetics system, which allows for selectively introducing individual mutations into the genome of an enterovirus. While extensively used in studying viral infection and vaccine development,^39−41^ reverse genetics has rarely been applied to understanding disinfection susceptibility. Two notable exceptions include an investigation of the sensitivity of murine norovirus to calcium hydroxide,^42^ as well as the sensitivity of PV1 to chlorine.^36^

Here, we employed a reverse genetic system for CVB5 and assessed how different amino acid substitutions in capsid proteins affects the virus’ sensitivity to free chlorine and heat. We thereby focused on ten amino acid substitutions between the chlorine-susceptible genogroup A and the more chlorine-resistant genogroup B that either locate on the capsid surface and hence provide good accessibility to chlorine or involve the highly chlorine-susceptible Met. A disinfection-tolerant mutant was structurally analyzed by cryo-electron microscopy (cryoEM), to provide a possible mechanism behind reduced disinfection sensitivity.

## Materials and Methods

### Cells

Buffalo green monkey kidney (BGMK) cells were kindly provided by the Spiez laboratory (Switzerland). The cells were grown in Eagles’s Minimum Essential Medium (MEM) supplemented with 10% of fetal bovine serum (FBS; Gibco) and 1% of penicillin–streptomycin (P/S; Gibco), and they were maintained in MEM supplemented with 2% FBS and 1% of P/S at 37 °C in humidified 5% CO_2_-saturated conditions.

### Construction of Infectious cDNA Clone

**Figure 1:**
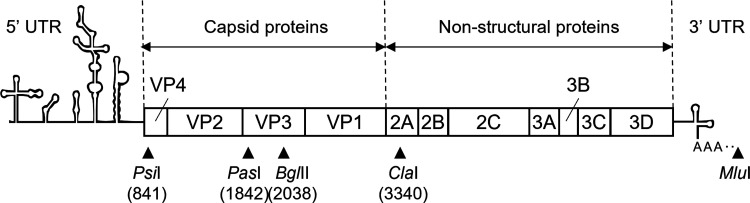
Genomic structure of CVB5F.cas (accession No. PP417940). An illustration of the genome organization is shown along with the position numbers of relevant restriction sites used to construct infectious cDNA clones in this study. The position number is based on CVB5 Faulkner (accession No. AF114383).

Plasmids containing partial CVB5 Faulkner (CVB5F) genome (Accession Number: AF114383) in a pUC57 vector were purchased (GenScript) and were cloned by Gibson Assembly^43^ using Gibson Assembly Mastermix (NEB) to produce an infectious, full-length cDNA clone of CVB5F. In this CVB5F clone (later termed CVB5F.cas), a *Cla*I restriction site (Figure 1) was introduced at a nonstructural protein region (nucleotide position 3340) by changing the sequence from ATAGAGTG to AT**C**GA**T**TG (position from 3339 to 3346) to generate a cassette vector.^40^ While this modification resulted in one amino acid change in the 2A protein (valine to leucine at amino acid position 17), the introduced substitution was accepted, as leucine 17 is present in the 2A protein of other enteroviruses, including E30 and E21.^40^ The whole sequence of the constructed clone, pCVB5F.cas, was deposited to GenBank under accession number PP417940.

### Mutagenesis

In addition to the CVB5F.cas clone, a total of 11 mutants were generated (Table 1). Ten of them were the mutants containing a single amino acid substitution and are referred to as CVB5F.cas.VP*n*.X*m*Y, where an amino acid at residue number *m* in the VP*n* region is replaced from X to Y, respectively. The other simultaneously contains all the unique amino acid substitutions from genogroup A to genogroup B (i.e., all the listed substitutions except for VP3.M63L in Table 1), termed CVB5F.cas.genogroupB. For generating CVB5F.cas.VP*n*.X*m*Y, pCVB5F.cas was site-directed mutagenized by polymerase chain reaction (PCR) using the primers listed in Table S1. A pair of *Psi*I, *Pas*I, *Bgl*II, and *Cla*I restriction sites was used to linearize pCVB5F.cas (Figure 1). The PCR-amplified region containing the desired mutation was cloned by Gibson Assembly to produce each infectious cDNA clone. For pCVB5F.cas.genogroupB, a pTwist Amp High Copy plasmid containing the genomic sequence of pCVB5F.cas.genogroupB flanked by *Psi*I and *Cla*I was purchased (Twist Bioscience). The regions flanked by the two restriction sites were amplified by PCR and cloned into pCVB5F.cas by Gibson Assembly. All the produced constructs were propagated in competent cells (5-α Competent *Escherichia coli*; NEB) and isolated by NucleoSpin Plasmid (TaKaRa). The introduced mutation was verified by Sanger sequencing.

**Table 1:** Substitutions of Amino Acid Residues Between Genogroups A and B of CVB5, and Description of Mutants Produced in this study.

			genogroup A		genogroup B				
protein	residue^a^	location^b^	(*n* = 7)	Faulkner	(*n* = 6)	at the capsid surface	Met invloved	mutants generated in this study	name of mutant^c^
VP1	19	interior	G	G	S				
VP1	95	interior	S	N	N				
VP1	156	EF loop	V	V	I	x		x	CVB5F.cas.VP1.V156I
VP1	180	buried, hydrophobic pocket	M	M	I		x	x	CVB5F.cas.VP1.M180I
VP1	276	C-terminal	D	D	E	x		x	CVB5F.cas.VP1.D276E
VP1	279	C-terminal	T	T	A	x		x	CVB5F.cas.VP1.T279A
VP2	37	interior	V	V	T				
VP2	45	interior	D	D	E				
VP2	137	EF loop (puff)	L	L	I	x		x	CVB5F.cas.VP2.L137I
VP2	156	EF loop (puff)	E	E	D	x		x	CVB5F.cas.VP2.E156D
VP2	160	EF loop (puff)	S	S	T	x		x	CVB5F.cas.VP2.S160T
VP2	260	C-terminal	K	K	R	x		x	CVB5F.cas.VP2.K260R
VP3	35	interior	E/N/D^d^	E	A				
VP3	63^e^	N-terminal helix close to β-strand B (knob)	M/L^d^	M	S/T^d^	x	x	x	CVB5F.cas.VP3.M63L
VP3	67^f^	N-terminal helix close to β-strand B (knob)	S/A	A	A				
VP3	88	CD loop	T/M/I^d^	T	I	x		x	CVB5F.cas.VP3.T88I
VP4	17	disordered	L	L	V				
VP4	45	interior	D	D	E				
VP4	47	interior	T	T	A				
VP1–4	all of the above except^e^,^f^								CVB5F.cas.genogroupB

a The numbering of amino acids is according to the residue positions in the CVB5 Faulkner.^63^

b The location was assigned according to previous studies.^40,64^

c CVB5F.cas corresponds to the Faulkner strain with a C*l*aI restriction site introduced at a non-structural protein region (nucleotide position 3340).

d Multiple amino acid residues were observed within the genogroup.

e Strains belonging to genogroup B do not possess single specific amino acids.

f The same amino acid residue is observed for CVB5 Faulkner and strains belonging to genogroup B.

### Generation of Viruses from Infectious cDNA Clones

A full-length CVB5 RNA was produced by *in vitro* transcription of an *MluI*-linearized plasmid using the T7 RiboMAX Express Large Scale RNA Production System (Promega). Then, 0.4–2.6 μg of the transcribed RNA was transfected into near-confluent BGMK cells prepared in a T25 flask using Lipofectamine MessengerMax transfection reagent (Thermo Scientific).^23^ After incubation at 37 °C in humidified 5% CO_2_-saturated conditions for 3 days, the transfected cells were frozen and thawed once and harvested. The cell suspension was centrifuged at 3500*g* for 15 min to pellet down cell debris. The supernatant was filtered through a 0.45 μm low protein binding durapore membrane (Merck Millipore Ltd.), and the progeny virus stock was aliquoted and stored at −20 °C until use.

A 100 μL aliquot of the progeny virus was passaged in a T150 flask with confluent BGMK cells for large-scale virus production. The passaged viral stock was recovered as described above. Then, 30 mL of the passaged virus stock was purified by sucrose-cushion ultracentrifugation followed by 0.22 μm membrane filtration as described previously.^22^ The purified viral stock was stored at 4 °C before use. Sanger sequence confirmed that the introduced mutation was preserved even after cell passage, except for CVB5F.cas.VP1.V156I. For this mutant, an ambiguous nucleotide, denoted Y (i.e., C or T), was observed at position 2914, but the mutation was synonymous, ensuring that the intended amino acid sequence was maintained (Table S2).

### Enumeration of Infectious Viruses

Infectious virus concentrations were enumerated by end point dilution assay using near-confluent BGMK cells maintained in 96-well plates as described elsewhere^9^ and were quantified according to the most probable number (MPN) method^44^ using the R package {MPN}.^45^ The lower limit of detection was 12 MPN mL^–1^ of the sample.

### Disinfection Experiments

#### Free Chlorine

The free chlorine disinfection experiments were conducted in a glass beaker in duplicate for each mutant. All experiments were conducted in a temperature-controlled room at 20 °C. A free chlorine working solution was prepared by diluting sodium hypochlorite (Reactolab SA, Switzerland) in 1 mM phosphate buffer (pH 7.0). The final free chlorine concentration in the working solution ranged from 0.53 to 0.62 mg L^–1^ as Cl_2_. The free chlorine concentration was measured by the *N*,*N*-diethyl-*p*-phenylenediamine (DPD) method^46^ using a DR300 Chlorine Pocket Colorimeter (Hach Company). This method yields results with a fold-change within ±5% compared to measurements obtained through direct photometry at 292 nm^47^ (ε_–OCl,__292nm_ = 350 M^–1^cm^–1^). Before each experiment, glass beakers were soaked with >50 mg L^–1^ of sodium hypochlorite overnight to quench the residual chlorine demand. The beakers were rinsed twice with Milli-Q water and once with the chlorine working solution. Then, 50 μL of virus stock solution was spiked into 11.5 mL of the working solution under constant stirring, to achieve a starting concentration of 4.1–5.2 log_10_ MPN mL^–1^. A 500 μL aliquot was collected every 30 or 45 s and mixed with 5 μL of 5000 mg L^–1^ sodium thiosulfate (Sigma-Aldrich, Germany) to instantly quench the residual free chlorine. A total of 3 time-series samples plus an untreated sample (i.e., sample at time zero), were taken. After the experiment, untreated and disinfected samples were stored at 4 °C for a maximum of 24 h prior to enumeration. The free chlorine concentration in the beaker was measured at the beginning and ten seconds after the collection of the last time-series sample. The decay in free chlorine concentration was less than 16% throughout each run. The chlorine exposure (CT value; concentration of free chlorine multiplied by contact time) for each sample was determined by integration of the time-dependent disinfectant concentration over exposure time, assuming first-order decay in free chlorine concentration over the course of the experiment. The inactivation rate constants (*k*) (mg^–1^ min^–1^ L) were determined based on the pooled data from duplicate experiments as the slope of–ln(*N/N*_0_) versus CT value by linear least-squares regression, where *N* is the infectious virus concentration at time *T* (MPN mL^–1^) and *N*_0_ is the infectious virus concentration at time 0 (MPN mL^–1^).

#### Heat

Heat treatment was conducted in a thermal cycler (GeneAmp PCR system 9700, Applied Biosystems) in triplicate. Five microliters of purified virus stock were spiked into thin-wall PCR tubes containing 45 μL of 1 mM phosphate buffer preheated at 50 °C and were incubated for 20 s. The condition for temperature and incubation time was selected because a similar condition was found to lead to an easily measurable level of inactivation for CVB5F.^33^ The incubated tubes were immediately cooled by placing them on crushed ice, and samples were stored at 4 °C for a maximum of 24 h. Inactivation was given by −log_10_(*N/N*_0_).

### CryoEM and Single Particle 3D Construction

#### Preparation of the Virus Sample for CryoEM Imaging

A 160 mL aliquot of the passaged virus stock of CVB5F.cas.genogroupB was placed on 20% sucrose cushion and ultracentrifuged at 150,000*g* for 3 h. After the supernatant was decanted, the pellet was resuspended with 500 μL of filtered phosphate-buffered saline (PBS;10 mM phosphate, 140 mM NaCl, 2.68 mM KCl, pH 7.4, Gibco). The suspension was again centrifuged at 10,000*g* for 3 min to remove the carry-over debris. The supernatant was collected and amended with paraformaldehyde at 100 μg mL^–1^ and incubated at 4 °C for 5 days to inactivate the samples. Paraformaldehyde chemically reacts with nucleic acids and proteins creating methylene cross-links between residues as well as introducing Schiff base and methylol group modifications,^48,49^ thereby leading to inactivation of the virus. This step was necessary to ensure that the virus sample can be handled and imaged under biosafety level 1 conditions. The samples were washed with PBS using an Amicon Ultra centrifugal unit (MWCO: 100 kDa, Merck Millipore) and subjected to size-exclusion chromatography to further purify the viral fraction as described elsewhere.^50^ Size-exclusion chromatography was performed using a HiPrep 16/60 Sephacryl S-500 HR column (Cytiva) running in tris-based buffer (25 mM tris-HCl, 150 mM NaCl, pH 7.5). Fractions corresponding to CVB5 were combined and concentrated to 4.5 mg mL^–1^ using Amicon Ultra centrifugal filter units with 100 kDa MWCO (Merck Millipore).

#### Grid Preparation and Imaging

Grids were prepared as described before.^50^ Briefly, 3 μL of the purified CVB5F.cas.genogroupB at 4.5 mg mL^–1^ concentration was loaded onto Quantifoil R 1.2/1.3 grids (EMS), which were previously glow-discharged for 30 s in a GloCube Plus device (Quorum Technologies). Grid vitrification was performed on a Vitrobot Mark IV with the following settings: Temperature = 10 °C; Humidity = 100%; Blotting force = 0; Wait time = 10 s; Blotting time varied in the 4–6 s range. Following the blotting step, the grids were plunge-frozen into liquid ethane, cooled by liquid nitrogen. Samples were imaged on a Glacios electron microscope (Thermo Fisher Scientific) equipped with an X-FEG electron source and operating at 200 kV voltage. Images were collected with a Falcon IVi camera in the electron-event-representation (EER) format. Nominal microscope magnification was set to 150,000 X resulting in a pixel size of 0.926 Å (at the specimen plane). Automated data collection was performed using the EPU software (Thermo Fisher Scientific). Data collection information is provided in Table S3.

#### Data Processing and Model Reconstruction

All data processing steps were performed in the cryoSPARC software package.^51^ Raw micrograph frames were aligned and dose-weighted using Patch Motion Correction while the CTF parameters were estimated using CTFFind.^52^ Particle picking was done using a combination of blob and template picker in cryoSPARC, resulting in 161 × 820 total extracted particles that were then subjected to two-dimensional (2D) classification. Particle classes that did not have any virus-resembling properties were removed, and virus-resembling particle classes were divided into empty and full, based on the absence/presence of internal viral components in the 2D classes. The “Empty” class comprised 75,212 particles (E). Particles containing internal viral components (∼28,000) were subjected to heterogeneous refinement with icosahedral symmetry imposed. The initial three-dimensional (3D) model was generated by Ab initio reconstruction of the full 2D-cleaned data set. Heterogeneous refinement resulted in 2 distinct subsets corresponding to the closed native virus state (F, 193 particles) and the intermediate-altered conformation (A, 27′505 particles). The F, A, and E subsets were then subjected to nonuniform refinement in cryoSPARC with icosahedral symmetry imposed and refinement of global and local CTF parameters. The resulting maps were used for the relaxation of the atomic models. The entire data processing workflow is shown in Figure S1. Model building and refinement were completed using a combination of manual steps in Coot^53^ and automated steps in Rosetta.^54^ Only 1 asymmetric unit was built per viral particle with icosahedral symmetry restraints imposed. Throughout the EM map, we observed additional densities surrounding the side chains of histidine and cysteine residues that cannot be assigned to any peptidic or posttranslational elements. We believe these to be the results of formaldehyde treatment,^55^ but we did not try to approximate them with atomic models. Model validation was performed in Phenix^56^ using the MolProbity^57^ and EMRinger^58^ metrics. The resulting models and maps were deposited in the protein data bank (PDB) and electron microscopy data bank (EMDB), respectively. Model statistics and PDB/EMDB accession numbers are shown in Table S4.

### Alignment of Amino Acid Sequences

A total of 112 exemplar virus isolates, each of which belong to a different genotype of human-infecting Enterovirus (i.e, Enterovirus A, B, C, and D) listed in Table S5, were identified from Virus Metadata Resource from International Committee on Taxonomy of Viruses.^59^ All the full-length amino acid sequences of polyproteins were aligned in Geneious Prime 2023.1.2 using MAFFT plugin^60^ with default settings. The alignments were examined for the amino acid variations at positions 180, 276, and 279 in the VP1 region.

### Statistical Analyses

All statistical analyses were performed using R version 4.3.0.^61^ Linear least-squares regression was performed with the lm function to estimate inactivation rate constants. An analysis of covariance (ANCOVA) with Dunnett’s or Tukey’s test was performed with the emtrends function in an R package {emmeans}. A one-way analysis of variance (ANOVA) with Dunnett’s or Tukey’s test was conducted with the R package {multcomp}.^62^

## Results

### Characterization of Engineered Mutants

**Figure 2:**
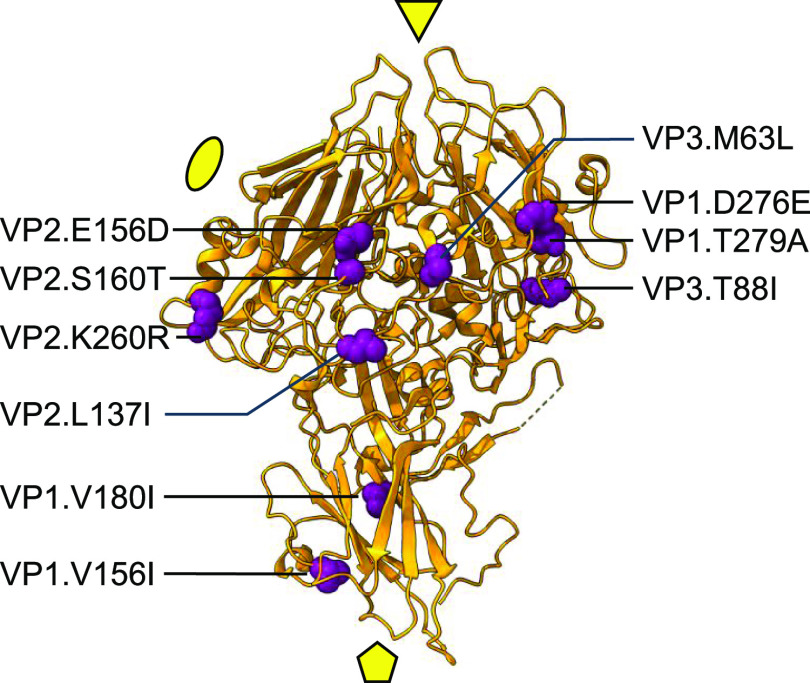
Location of the ten amino acid residues presented using atomic sphere representation (purple) on the published structure of the CVB5 (PDB ID: 7C9Y; orange). For simplicity, only 1 protomer is shown with the locations of 2-, 3-, and 5-fold symmetry axes indicated with ellipse, triangle, and pentamer, respectively.

To identify the amino acid changes between genogroups A and B, the amino acid sequences in the capsid proteins (i.e., VP1-VP4) of previously tested CVB5 variants (Accession No: MW015045 - MW015056, and AF114383)^28^ were aligned. Of these variants, seven belong to genogroup A and six belong to genogroup B (Table 1). In the capsid protein region, a total of 13 conserved changes were observed between the two genogroups, and six additional changes were found to be common in all except for one variant. Six amino acid substitutions are found in VP1, six in VP2, four in VP3, and three in VP4. Of these, ten were deemed of high interest (Figure 2). Specifically, nine substitutions, including VP1.V156I, VP1.D276E, VP1.T279A, VP2.L137I, VP2.E156D, VP2.S160T, VP2.K260R, VP3.M63L, and VP3.T88I, are located on the capsid surface and are hence easily accessible to free chlorine. Among them, VP3.M63L involves the substitution of Met with a more chemically stable amino acid. An additional such substitution is found at a position buried within the VP1 protein, VP1.M180I. These last two amino acid substitutions were hypothesized to be the most effective in reducing sensitivity to free chlorine.

To investigate the effect of the ten selected amino acid substitutions, we engineered a total of 12 mutants. We first constructed the clone CVB5F.cas, which served as the control strain and which differed from the Faulkner strain only by a single residue in the nonstructural region, see Materials and Methods. We then constructed ten infectious cDNA clones harboring each critical amino acid substitution individually. Finally, a construct harboring all of the genogroup B-specific amino acid substitutions in the capsid proteins, which the CVB5 Faulkner strain does not possess, was prepared (see Table 1). All constructs successfully produced infectious progeny viruses at comparable titers, with the final concentration of purified virus stocks ranging from 6.5 to 7.6 log_10_ MPN mL^–1^.

### Sensitivity to Free Chlorine

**Figure 3:**
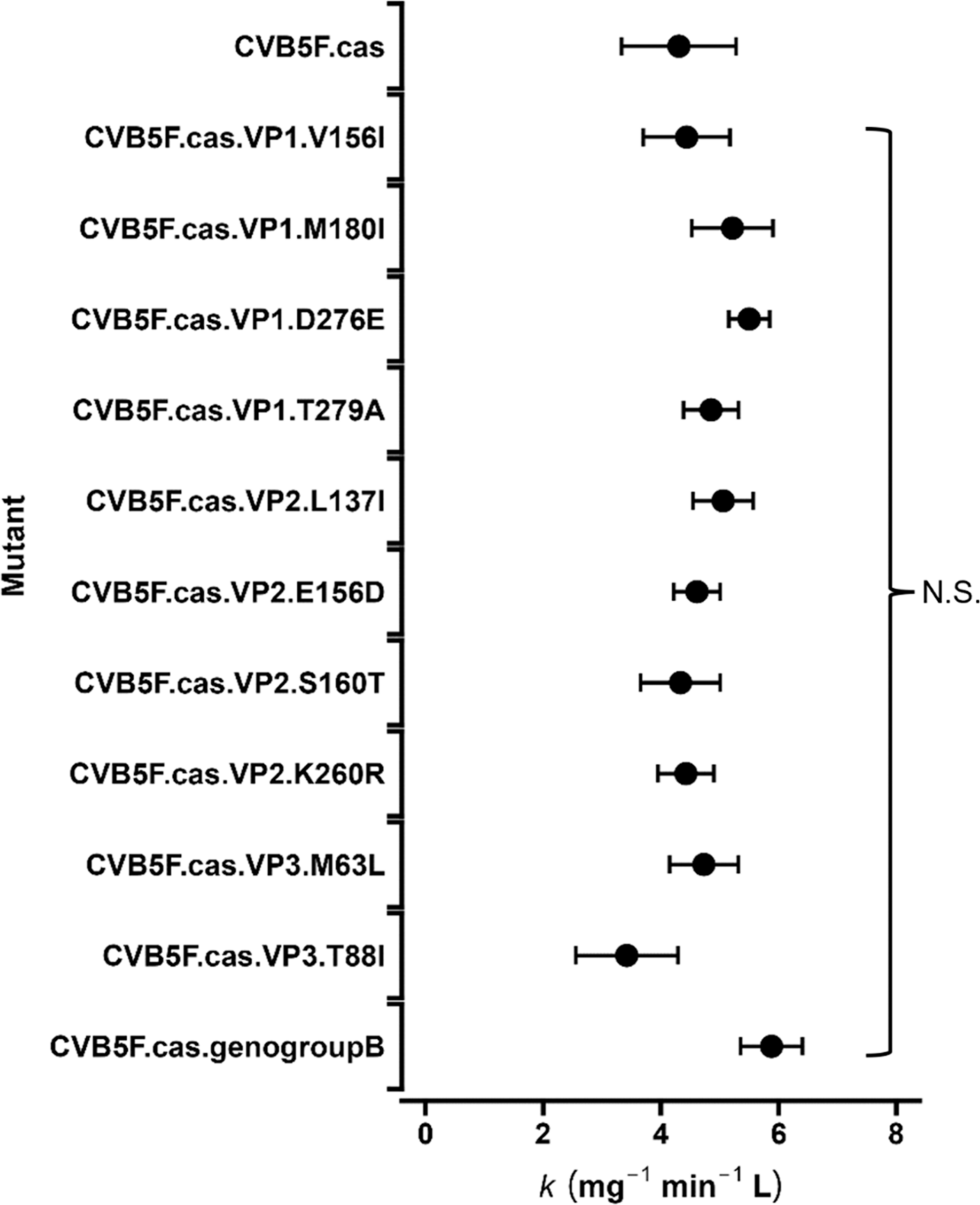
Inactivation rate constants for each mutant by free chlorine. Error bars indicate the standard error on the rate constant, based on pooled duplicate experiments. ANCOVA with Dunnett’s test suggests that the rate constants are not statistically different between CVB5F.cas and each mutant. N.S. = not significant.

Generated mutants were tested for chlorine sensitivity by measuring inactivation curves in bench-scale experiments. Experimental data for free chlorine inactivation are provided in Figure S2. Estimated inactivation rate constants are shown in Figure 3. The inactivation rate constant for CVB5F.cas was 4.3 mg^–1^ min^–1^ L. The rate constants for other mutants ranged from 3.4 to 5.9 mg^–1^ min^–1^ L. The fold-change in rate constants between CVB5F.cas and each mutant ranged from 0.7 to 1.3, which is less than the 1.9-fold change between environmental isolates belonging to genogroup A and B reported in our past study.^28^ None of the mutants, including those substituting Met and the one harboring all the genogroup B-specific amino acid substitutions in the capsid proteins, exhibited a significantly different inactivation rate constant compared to CVB5F.cas (*P* > 0.05, ANCOVA with Dunnett’s test). Moreover, the rate constants were not significantly different among any pairs of mutants (*P* > 0.05, ANCOVA with Tukey’s test). These results suggest that the introduced amino acid substitutions are not responsible for the reduced chlorine sensitivity of CVB5 in genogroup B.

### Sensitivity to Heat

**Figure 4:**
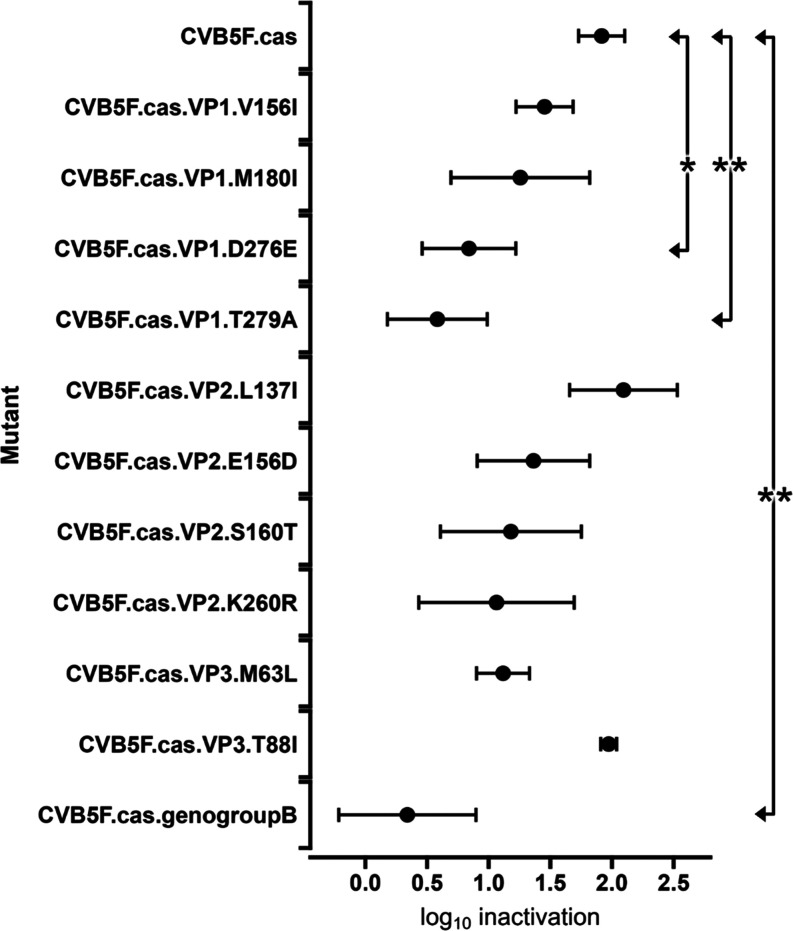
Inactivation of each mutant by heat treatment at 50 °C for 20 s. Log_10_ inactivation is shown as the mean ± the standard deviation of three replicates. A one-way ANOVA with Dunnett’s test was performed to compare CVB5F.cas with each mutant. The double arrows point to mutant pairs that exhibit a significant difference. (**P < 0.01, *P < 0.05).

The mutants were tested for heat sensitivity by exposure to 50 °C for 20 s. Experimental inactivation data of heat treatment is provided in Figure 4. The inactivation of CVB5F.cas was 1.9 ± 0.2 log_10_, with a range of 0.34 ± 0.56 to 2.1 ± 0.4 log_10_ for the other mutants. Significantly lower inactivation was observed for CVB5F.cas.VP1.D276E and CVB5F.cas.VP1.T279A compared to CVB5F.cas. (ANOVA with Dunnett’s test; *P* < 0.05). The inactivation of CVB5F.cas.genogroupB, which includes the two aforementioned substitutions, was also significantly lower (ANOVA with Dunnett’s posthoc analysis; *P* < 0.01). Among mutant pairs, CVB5F.cas.genogroupB and CVB5F.cas.VP1.T279A were also significantly less heat sensitive compared with CVB5F.cas.VP2.L137I and CVB5F.cas.VP3.T88I (ANOVA with Tukey’s test; *P* < 0.05). These results are consistent with previous data reporting that all the CVB5 variants containing substitutions VP1.D276E and VP1.T279A exhibited lower heat sensitivity compared to CVB5F.^7^

**Figure 5:**
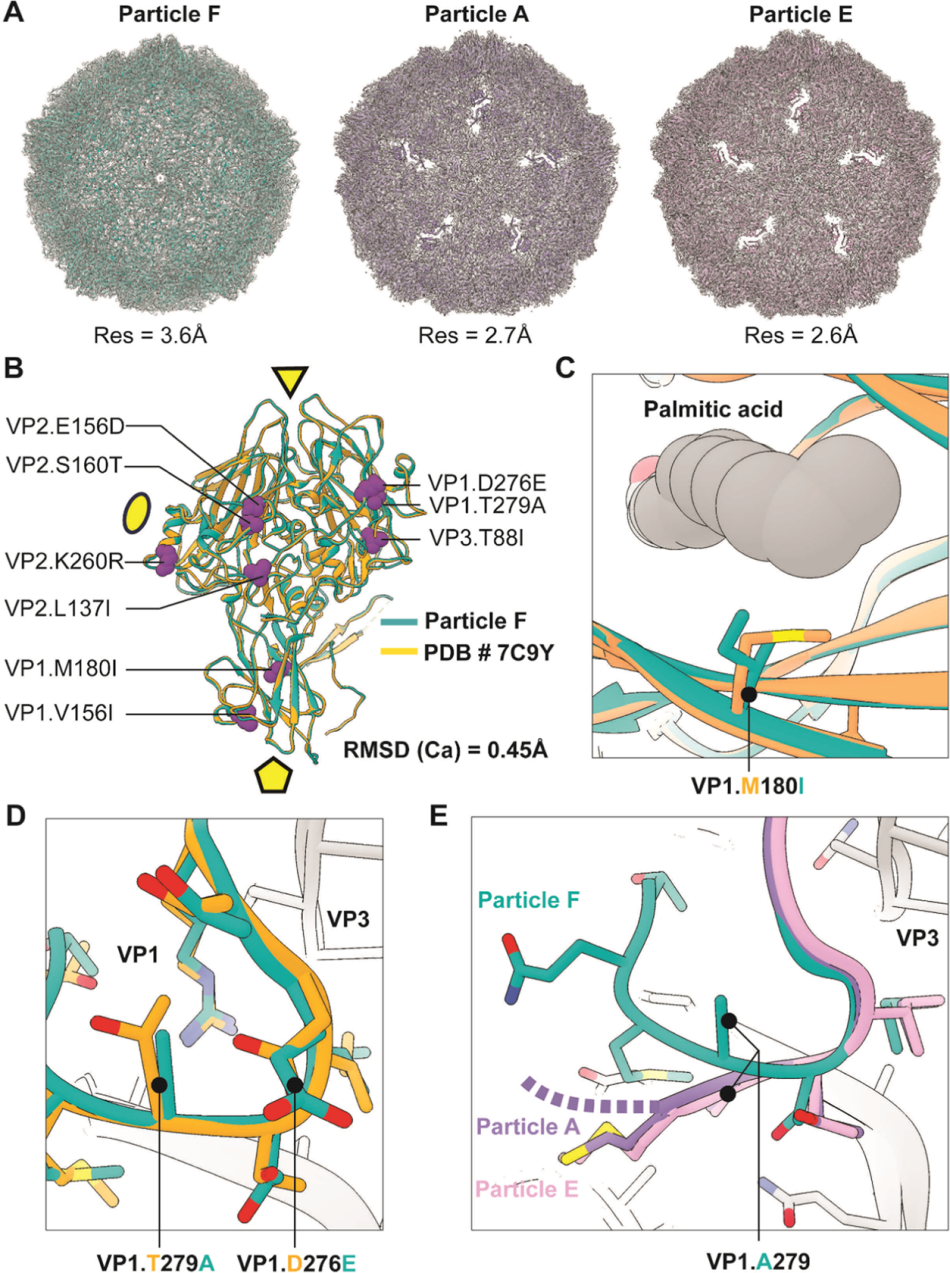
Structural Characterization of CVB5F.cas.genogroupB by cryoEM. (A) Reconstructed 3D maps and models of the capsids corresponding to mature closed virion (F), intermediated-altered state (A), and empty viral capsid (E). The maps are presented as transparent gray surface and cartoon representation was used for atomic models. (B) Overlay of the CVB5F.cas.genogroupB (F) model (turquoise) and the previously published structure of the CVB5 (PDB ID: 7C9Y; orange) with the mutated residues presented using atomic sphere representation (magenta). For simplicity, only 1 protomer is shown with the locations of 2-, 3-, and 5-fold symmetry axes indicated with ellipse, triangle, and pentamer, respectively. (C) Close-up view of the pocket in CVB5F.cas.genogroupB and nonmutated CVB5 structure with the palmitate shown in gray (sphere representation) and the alternative VP1.180 residues indicated. (D) Enlarged view of the VP1 C-terminal loop with the locations of VP1.279 and VP1.276 mutation sites indicated. The same color scheme used as in panel B. (E) Altered conformations of the structured part of VP1 C-terminus in different reconstructed states of the CVB5F.cas.genogroupB. Note the alternative conformation of the VP1.A279 residue.

To elucidate the mechanism behind reduced heat sensitivity of the CVB5F.cas.genogroupB mutant, we subjected it to analysis by cryoEM. Viruses were inactivated by formaldehyde treatment and imaged as described in the Materials and Methods. Data collection statistics are shown in Table S3, and the data processing workflow is illustrated in Figure S1. We applied a combination of 2D and 3D classification steps in cryoSPARC package^51^ to separate the particles corresponding to mature virions (F), intermediate-altered state (A), and empty viral capsids (E). These conformational states are commonly resolved in cryoEM analyses of enteroviruses,^64^ with A and E corresponding to the expanded viral particles with or without the internal viral components (nonstructural proteins and genetic material). The resulting EM density maps were at 3.6, 2.7, and 2.6 Å global resolution for the capsid-corresponding part of particles F, A, and E, respectively, thus allowing building of atomic models of each state (Figure 5A and Table S4).

Surprisingly, only ∼0.2% of the total data set corresponded to mature virions in the F state (193 particles). We believe this to be a result of formaldehyde inactivation. Nevertheless, the reconstructed atomic model closely matched the previously published CVB5 structure based on genogroup A (Figure 5B; and a previous study^64^). The root-mean-square deviation (RMSD) of Cα positions between the two structures was 0.45 Å. This is consistent with the fact that most mutations were relatively conservative (L → I, E → D, S → T, K → R) and unlikely to induce major structural rearrangements. We then analyzed the locations of genogroup B mutations and if any local conformational changes arise due to their presence. None of the mutations were located at the interfaces of multiple protomers (each comprising a single copy of VP1–4). Therefore, we excluded the possibility of increased thermotolerance being due to stronger interprotomer interactions and proposed intraprotomer stabilization as a more probable mechanism.

We first focused on the VP1.M180I mutation located in the hydrophobic pocket, a region that has been implicated in enhanced thermotolerance.^36,37^ Amino acids at this position make direct contact with the noncovalently bound palmitate residue (i.e., the pocket factor; Figure 5C). The pocket factor is essential for the proper assembly of the receptor binding domain and is released during the viral entry process. While we noticed somewhat weaker density for the pocket factor compared to the previously published cryoEM map of CVB5 F-particle (EMD-30321^64^), the switch from M to I does not alter the size of the hydrophobic pocket, and these two amino acids are commonly found at position 180 across the *Enterovirus* genus relevant to human infection, including 112 exemplar strains of each genotype of *Enterovirus A, B, C*, and *D* (Table S5), listed in Virus Metadata Resource.^59^ This is consistent with our findings that the heat sensitivity of CVB5F.cas.VP1.M180I is not significantly different from that of CVB5F.cas.

The two amino acid substitutions causing the largest perturbation in relative heat sensitivity, VP1.D276E, and VP1.T279A, are located at the C-terminus of VP1. These two residues interact with each other and contribute to the external part of the VP1:VP3 interface, both through direct contact and indirectly by stabilization of the local loop region comprising residues VP1.E272-T282 (Figure 5D). The VP1.D276E and VP1.T279A mutations do not seem to result in any significant conformational change compared to the unmutated CVB5 variant (PDB ID: 7C9Y), and it is not evident how each mutation alone leads to the observed enhnaced tolerance toward heat (Figure 4). However, their pairing could affect local molecular packing and influence the dynamics of viral unfolding during infection or thermal inactivation. Consistently, this region in VP1 undergoes partial restructuring in intermediate-altered (A) and empty particle (E) states (Figure 5E), with alanine at VP1.279 facing toward the hydrophobic pocket in VP3 assembled by residues VP3.P86, VP3.A141 and VP3.Y189. Although the C-terminus of VP1 is flexible in different enterovirus structures, the resulting conformation in A and E particles of CVB5F.cas.genogroupB is distinct from the previously published reconstruction of CVB5-A and E particles having the original VP1.D276/VP1.T279 pairing (Figure S3 and the previous study^64^), supporting that VP1.D276E and VP1.T279A mutations may affect the assembly and local dynamics of the VP1:VP3 interface.

Several other areas in particle A and E states of CVB5F.cas.genogroupB also exhibit different conformations and/or greater flexibility compared with corresponding CVB5 structures from the PDB (Figure S3). Most prominent changes were in the N-terminal region of VP1 (VP1.Q49-S60), VP2 (VP2.L42-Q52), and VP3 (I168–V181). While these discrepancies could indicate altered energy landscape of intermediate viral conformations in CVB5F.cas.genogroupB, none of the listed residue ranges contain genogroupB mutations or create direct contact with them. Therefore, the observed effects are either indirectly influenced by amino-acid substitutions, or potentially a consequence of formaldehyde treatment.

Altogether, based on structural analysis of the CVB5F.cas.genogroupB we propose that mutations conferring greater tolerance to thermal inactivation by perturbing local molecular interactions which may lead to improved stability of infectious virions (F) and altered conformations of uncoating intermediates (e.g., particles A and E).

## Discussion

The impact of mutations on disinfection sensitivities has been a subject of debate; yet, the direct effect of mutations has been rarely evaluated. Given that CVB5 is among the most resistant viruses to common disinfectants, our study employed a reverse genetics system to assess how single amino acid substitutions influence the sensitivity of this virus to free chlorine and heat, yielding several important insights.

Our free chlorine disinfection experiments showed a surprising result; the substitution of Met by aliphatic ones did not coincide with a lowered sensitivity to free chlorine. This finding contradicts hypotheses raised by prior studies on the role of oxidizable residues in free chlorine disinfection,^7,26,28,65,66^ but is consistent with reports on the absence of chlorine resistance in PV1 following a Met to Val substitution in VP1.^36^ The sensitivity to other oxidants, such as peracetic acid are also believed to be governed by the structure and protein compositions of viruses.^27,29^ Our result with free chlorine implies a need to revisit this hypothesis.

We then sought alternative explanations for genogroup-dependent CVB5 sensitivity to free chlorine. Past studies showed that another oxidant, chlorine dioxide, primarily damages the viral genome region spanning approximately from nucleotide 1 to 120, within 5′ untranslated region (5′UTR), leading to inactivation of PV1 and enterovirus 71.^67^ It was also reported that the 5′UTR of hepatitis A virus is the most degraded by free chlorine across the whole genome.^68^ The 5′ UTR contains a cloverleaf structure directing viral RNA replication and an internal ribosome entry site that initiates translation.^69^ This suggests that the region is also essential for enterovirus infectivity.

Interestingly, a 5′UTR-based classification of the CVB5 isolates used in our previous work^28^ segregated the variants into the same clusters as the VP1-based classification, with the sole exception of CVB5 Faulkner (Figure S4). In contrast, when other genome regions were used as classification bases, the clustering diverged (Table S6). Moreover, the inactivation rate constants of the variants that we previously tested are also significantly different between the two 5′UTR-based genogroups (Wilcoxon-rank sum test: *P* < 0.01). Given that the genome damage induced by free chlorine also contributes to viral inactivation,^13,70^ it is plausible that the mutations in the 5′UTR alter the composition and the secondary structures, thereby changing chlorine sensitivity. A total of 12 common mutations were observed among the two 5′UTR-based genogroups. To confirm the role of these mutations, future studies should test the chlorine sensitivity of a mutant that carries all of the 5′UTR mutations and test its sensitivity to free chlorine and subsequently focus on the role of each individual mutation.

Our study furthermore found that the heat sensitivity of CVB5 is lowered with the amino acid substitutions in the C-terminal region of VP1. This highlights the importance of characterizing the thermostability of a given viral genotype or species based on multiple variants rather than a single strain.

Although unveiling the exact mechanism of the reduced heat sensitivity needs further examination of the uncoating mechanism of CVB5, we speculate a contribution of the stabilized VP1:VP3 interface by the substitution of VP1.D276E and VP1.T279A. A past structural analysis of coxsackievirus A7 showed that the uncoating triggered by heat treatment at 56 °C results in the rotation of VP1, which causes major conformational changes at the interfaces of the capsid proteins VP1, VP2, and VP3.^71^ The substitution of VP1.D276E and VP1.T279A may thus minimize the dissociation of the VP1:VP3 interface by heat treatment, resulting in reduced heat sensitivity. Interestingly, an alignment analysis of 112 exemplar strains of the *Enterovirus* genus, representing all genotypes relevant to human infection, revealed no occurrence of the combination of VP1.E276 and VP1.A279 observed in CVB5 genogroup B, whereas the combination of VP1.D276 and VP1.T279 observed in CVB5 genogroup A is also observed for 15 other *Enterovirus* genotypes (e,g, PV1, CVA9, CVA1). Therefore, the reduced heat sensitivity by this combination of amino acid substitution is assumed to be specific to CVB5 genogroup B. It is worth investigating the relationship between the amino acid substitution at the C-terminal VP1 region, its effect on the VP1:VP3 interface, and heat sensitivity of viruses in further studies. Moreover, further studies need to investigate the thermotolerance under higher temperatures, where a different mechanism of thermal inactivation may occur.^72^

This study also suggests that the chlorine and thermotolerance of CVB5 do not necessarily correlate, at least if thermotolerance is induced by amino acid substitutions in the capsid proteins. This is consistent with previous studies that found no effect of thermotolerance-inducing substitutions in the capsid protein of PV1 on chlorine resistance.^36^ Instead, the two effects appear to be induced by separate sets of amino acid substitutions that co-occur in CVB5 isolates belonging to genogroup B. Nevertheless, a correlation between chlorine and thermotolerance cannot be excluded for mutations occurring in other regions of the genome rather than the capsid proteins region. In particular, future works should focus on the role of mutations in the 5′ UTR on both chlorine and heat treatment.

Finally, the employed reconstituted infectious cDNA clone features a cassette vector system where capsid protein regions can be substituted with that from a different strain, genogroup, or genotype.^73^ Testing these viruses allows for investigating the fundamental cause of variant-, genogroup-, and genotype-dependent disinfection sensitivity of the enterovirus. The use of reverse genetics presents significant potential for advancing our understanding of the differing sensitivities of viruses to disinfectants and their respective inactivation mechanisms.

## Data Availability

All data discussed in this manuscript are accessible via 10.5281/zenodo.10141286. 3D maps and models from the electron microscopy experiments have been deposited to the Electron Microscopy Data Bank (http://www.emdatabank.org/) and the Protein Data Bank (http://www.rcsb.org/), respectively. The accession numbers are listed in the Material and Methods Section and Table S4.

## Supporting Information Available

Details of site-directed mutagenesis and cryoEM; Sanger sequencing of propagated variants; list of exemplar isolates of human enterovirus; phylogenetic analysis of previously tested variants; workflow used to process cryoEM data; structural analysis of CVB5F.cas.genogroupB; and inactivation curves for free chlorine disinfection experiments (PDF)
