# Supplementary material for: Influence of Amino Acid Substitutions in Capsid Proteins of Coxsackievirus B5 on Free Chlorine and Thermal Inactivation
Source: Environ Sci Technol. 2024 Mar 15;58(12):5279–89. doi: 10.1021/acs.est.3c10409 (PMC10976892; doi:10.1021/acs.est.3c10409)
Supplement: Supplementary file 1 — es3c10409_si_001.pdf [file es3c10409_si_001.pdf]

SUPPORTING INFORMATION

Influence of amino acid substitutions in capsid proteins of coxsackievirus B5 on free chlorine and thermal inactivation

Shotaro Torii[a], Jérôme Gouttenoire[b], Kiruthika Kumar[c], Aleksandar Antanasijevic[c], Tamar Kohn[a*]

[a] Laboratory of Environmental Chemistry, School of Architecture, Civil and Environmental Engineering (ENAC), École Polytechnique Fédérale de Lausanne (EPFL), CH-1015 Lausanne, Switzerland
[b] Division of Gastroenterology and Hepatology, Lausanne University Hospital and University of Lausanne, CH-1011 Lausanne, Switzerland
[c] Virology and Structural Immunology Laboratory, School of Life Sciences, École Polytechnique Fédérale de Lausanne (EPFL), CH-1015 Lausanne, Switzerland

*Corresponding author: Tamar Kohn
Email address: tamar.kohn@epfl.ch

Contents (fifteen pages, six Tables, and four Figures)

Tables

Table S1 Primers used for site-directed mutagenesis.
Table S2 Results of Sanger sequencing.
Table S3 CryoEM data collection information
Table S4 Model building and refinement information
Table S5 List of exemplar virus isolates.
Table S6 Phylogenetic classification of previously tested CVB5 variants

Figures

Figure S1 Workflow to process cryoEM data.
Figure S2 Inactivation curves for free chlorine.
Figure S3 Comparison CVB5.genogroupB.cas particles with previously published structures.
Figure S4 Phylogenetic tree constructed for 5’ UTR region of CVB5.

Table S1 Primers used for site-directed mutagenesis.

**Name**	**Sequence (5' - 3')**
VP2.L137I-fd	GGG TGC GCG ACG ATA GCA AAC AAA CCT GA
VP2.L137I-rv	TCA GGT TTG TTT GCT ATC GTC GCG CAC CC
VP2.E156D-fd	CGC CAA CGT GTT TGA CTC TCA GAA CTC TTC
VP2.E156D-rv	GAA GAG TTC TGA GAG TCA AAC ACG TTG GCG
VP2.S160T-fd	TTG AGT CTC AGA ACA CGT CGG GAC AAA CAG
VP2.S160T-rv	CTG TTT GTC CCG ACG TGT TCT GAG ACT CAA
VP2.K260R-fd	CGC TTG GCT GGC AGA CAG GGG CTT CCC ACA
VP2.K260R-rv	TGT GGG AAG CCC CTG TCT GCC AGC CAA GCG
VP3.M63L-fd	CTG AGG GGA AGG TGC TCT CCA TTG AAG CCT A
VP3.M63L-rv	TAG GCT TCA ATG GAG AGC ACC TTC CCC TCA G
VP3.T88I-fd	GGA TTC CCA CTG ATC CCA GGG GCT AGT AG
VP3.T88I-rv	CTA CTA GCC CCT GGG ATC AGT GGG AAT CC
VP1.V156I-fd	CGT GCC CAC AAA AAT CAA CAG CTA CAG TTG
VP1.V156I-rv	CAA CTG TAG CTG TTG ATT TTT GTG GGC ACG
VP1.M180I-fd	TGC CCC ACC TCG GAT CTC GAT ACC CTT CAT TAG
VP1.M180I-rv	CTA ATG AAG GGT ATC GAG ATC CGA GGT GGG GCA
VP1.D276E-fd	GAG GGC AGA ACA GAG ATA ACG ACC ATG C
VP1.D276E-rv	GCA TGG TCG TTA TCT CTG TTC TGC CCT C
VP1.T279A-fd	GAA CAG ACA TAA CGG CTA TGC AAA CCA CTG
VP1.T279A-rv	CAG TGG TTT GCA TAG CCG TTA TGT CTG TTC

Table S2 Confirmation of the introduced mutation by Sanger sequencing.

**Mutant**	**Position of the amino acid residue (nt)**	**Sequence of the corresponding residue**	
		**CVB5F.cas**	**Mutant**
CVB5F.cas.VP2.L137I	1358-1360	CTG	ATA
CVB5F.cas.VP2.E156D	1415-1717	GAG	GAC
CVB5F.cas.VP2.S160T	1427-1429	TCT	ACG
CVB5F.cas.VP2.K260R	1727-1729	AAA	AGA
CVB5F.cas.VP3.M63L	1919-1921	ATG	CTC
CVB5F.cas.VP3.T88I	1994-1996	ACT	ATC
CVB5F.cas.VP1.V156I	2912-2914	GTA	ATY[a]
CVB5F.cas.VP1.M180I	2984-2986	ATG	ATC
CVB5F.cas.VP1.D276E	3272-3274	GAC	GAG
CVB5F.cas.VP1.T279A	3281-3283	ACC	GCT
CVB5F.cas.genogroupB [b]	-	-	-

[a] Y represents pyrimidine (C or T). Both ATC and ATT codes isoleucine(I).

[b] All the substitution was successfully introduced.

Table S3 CryoEM data collection information

	**CVB5F.cas.genogroupB**
**Microscope**	TFS Glacios (X-FEG)
**Voltage (kV)**	200
**Detector**	Falcon IVi
**Recording mode**	EER
**Nominal Magnification**	150,000 X
**Movie micrograph pixel size**	0.926
**Dose rate (e^{-}/Å^{2}/s)**	9.0
**Total Exposure Dose (e^{-}/Å^{2})**	40
**Number of EER fractions**	40
**Movie micrograph exposure time (s)**	4.4
**Nominal under focus range (µm)**	0.8 – 1.0
**Number of movie micrographs**	6653

Table S4 Model building and refinement information

	F-particle	A-particle	E-particle
**EMDB ID**	EMD-18942	EMD-18943	EMD-18944
**Map Resolution (Å)**	3.6	2.7	2.6
**Map Symmetry**	I	I	I
**PDB ID**	8R5X	8R5Y	8R5Z
**Residues**	819	658	658
**Amino-acids**	818	658	658
**Ligands**	1	0	0
**RMSD Bonds**	0.021	0.020	0.019
**RMSD Angles**	1.741	1.685	1.718
**Ramachandran**			
**Outliers (%)**	0.00	0.00	0.00
**Allowed (%)**	1.11	0.78	1.09
**Favored (%)**	98.89	99.22	98.91
**Rotamer outliers**	0.00	0.00	0.00
**Clash score**	1.20	1.98	1.29
**Molprobity score**	0.84	0.96	0.85
**EMRinger score**	3.09	5.22	4.83

Table S5 List of exemplar virus isolates extracted from Virus Metadata Resource from International Committee on Taxonomy of Viruses[1].

**Species**	**Genotype(s)**	**Virus name abbreviation(s)**	**Virus isolate designation**	**Virus GENBANK accession**
Enterovirus A	coxsackievirus A2	CVA2	Fleetwood (Delaware/47)	AY421760
Enterovirus A	coxsackievirus A3	CVA3	Olson (New York/48)	AY421761
Enterovirus A	coxsackievirus A4	CVA4	High Point (North Carolina/50)	AY421762
Enterovirus A	coxsackievirus A5	CVA5	Swartz (New York/50)	AY421763
Enterovirus A	coxsackievirus A6	CVA6	Gdula	AY421764
Enterovirus A	coxsackievirus A7	CVA7	Parker	AY421765
Enterovirus A	coxsackievirus A8	CVA8	Donovan (New York/49)	AY421766
Enterovirus A	coxsackievirus A10	CVA10	Kowalik (New York/50)	AY421767
Enterovirus A	coxsackievirus A12	CVA12	Texas 12 (Texas/48)	AY421768
Enterovirus A	coxsackievirus A14	CVA14	G-14 (South Africa/50)	AY421769
Enterovirus A	coxsackievirus A16	CVA16	G-10 (South Africa/51)	U05876
Enterovirus A	enterovirus A71	EV-A71	BrCr	U22521
Enterovirus A	enterovirus A76	EV-A76	FRA91-10369	AY697458
Enterovirus A	enterovirus A89	EV-A89	BAN00-10359	AY697459
Enterovirus A	enterovirus A90	EV-A90	BAN99-10399	AY697460
Enterovirus A	enterovirus A91	EV-A91	BAN00-10406	AY697461
Enterovirus A	enterovirus A92	EV-A92	USA/GA99/RJg-7	EF667344
Enterovirus A	enterovirus A114	EV-A114	V13-0285/IND/2013	KU355876
Enterovirus A	enterovirus A120	EV-A120	MAD-2741-11 (Madagascar)	LK021688
Enterovirus A	enterovirus A121	EV-A121	V13-0682/IND/2013	KU355877
Enterovirus A	enterovirus A122; simian virus 19	EV-A122	M19s	AF326754
Enterovirus A	enterovirus A123; simian virus 43	EV-A123	OM112t	AF326761
Enterovirus A	enterovirus A124; simian virus 46	EV-A124	OM22	AF326764
Enterovirus A	enterovirus A125; baboon enterovirus A13	EV-A125	A13	AF326750
Enterovirus B	coxsackievirus B3	CVB3	Nancy (Connecticut/US/49)	M88483
Enterovirus B	coxsackievirus B1	CVB1	Japan	M16560
Enterovirus B	coxsackievirus B2	CVB2	Ohio-1 (Ohio/US/47)	AF081485
Enterovirus B	coxsackievirus B4	CVB4	JVB (New York/US/51) (Benschoten)	X05690
Enterovirus B	coxsackievirus B5	CVB5	Faulkner (Kentucky/US/52)	AF114383

Enterovirus B	coxsackievirus B6	CVB6	Schmitt (Philippines/53) (1-15-21)	AF039205
Enterovirus B	coxsackievirus A9	CVA9	Griggs	D00627
Enterovirus B	echovirus 1	E1	Farouk (Egypt/51)	AF029859
Enterovirus B	echovirus 2	E2	Cornelis (Connecticut/US/51)	AY302545
Enterovirus B	echovirus 3	E3	Morrisey (Connecticut/US/51)	AY302553
Enterovirus B	echovirus 4	E4	Pesacek (Connecticut/US/51)	AY302557
Enterovirus B	echovirus 5	E5	Noyce (Maine/54)	AF083069
Enterovirus B	echovirus 6	E6	D'Amori (Rhode Island/55)	AY302558
Enterovirus B	echovirus 7	E7	Wallace (Ohio)	AY302559
Enterovirus B	echovirus 9	E9	Hill (Ohio/US/53)	X84981
Enterovirus B	echovirus 11	E11	Gregory (Ohio)	X80059
Enterovirus B	echovirus 12	E12	Travis (Philippines/53)	X79047
Enterovirus B	echovirus 13	E13	Del Carmen (Philippines/53)	AY302539
Enterovirus B	echovirus 14	E14	Tow (Rhode Island/54)	AY302540
Enterovirus B	echovirus 15	E15	Ch 96-51 (Charleston) (West Virginia/51)	AY302541
Enterovirus B	echovirus 16	E16	Harrington (Massachusetts/51)	AY302542
Enterovirus B	echovirus 17	E17	CHHE-29 (Mexico City)	AY302543
Enterovirus B	echovirus 18	E18	Metcalf (Ohio)	AF317694
Enterovirus B	echovirus 19	E19	Burke (Ohio)	AY302544
Enterovirus B	echovirus 20	E20	JV-1 (Washington DC/55)	AY302546
Enterovirus B	echovirus 21	E21	Farina (E26D) (Massachusetts/50)	AY302547
Enterovirus B	echovirus 24	E24	DeCamp (Ohio/56)	AY302548
Enterovirus B	echovirus 25	E25	JV-4 (Washington DC/57)	AY302549
Enterovirus B	echovirus 26	E26	Coronel (11-3-6) (Philippines/53)	AY302550
Enterovirus B	echovirus 27	E27	Bacon (1-36-4) (Philippines/53)	AY302551
Enterovirus B	echovirus 29	E29	JV-10 (Washington DC/55)	AY302552
Enterovirus B	echovirus 30	E30	Bastianni (New York/58)	AF162711
Enterovirus B	echovirus 31	E31	Caldwell (Kansas/55)	AY302554
Enterovirus B	echovirus 32	E32	PR-10 (Puerto Rico)	AY302555
Enterovirus B	echovirus 33	E33	Toluca-3 (Mexico/59)	AY302556
Enterovirus B	enterovirus B69	EV-B69	Toluca-1 (Mexico/59)	AY302560
Enterovirus B	enterovirus B73	EV-B73	CA55-1988	AF241359
Enterovirus B	enterovirus B74	EV-B74	USA/CA75-10213	AY556057
Enterovirus B	enterovirus B75	EV-B75	USA/OK85-10362	AY556070

Enterovirus B	enterovirus B77	EV-B77	CF496-99	AJ493062
Enterovirus B	enterovirus B79	EV-B79	USA/CA79-10384	AY843297
Enterovirus B	enterovirus B80	EV-B80	USA/CA67-10387	AY843298
Enterovirus B	enterovirus B81	EV-B81	USA/CA68-10389	AY843299
Enterovirus B	enterovirus B82	EV-B82	USA/CA64-10390	AY843300
Enterovirus B	enterovirus B83	EV-B83	USA/CA76-10392	AY843301
Enterovirus B	enterovirus B84	EV-B84	CIV2003-10603	DQ902712
Enterovirus B	enterovirus B85	EV-B85	BAN00-10353	AY843303
Enterovirus B	enterovirus B86	EV-B86	BAN00-10354	AY843304
Enterovirus B	enterovirus B87	EV-B87	BAN00-10396	AY843305
Enterovirus B	enterovirus B88	EV-B88	BAN01-10398	AY843306
Enterovirus B	enterovirus B97	EV-B97	BAN99-10355	AY843307
Enterovirus B	enterovirus B98	EV-B98	T92-1499	AB426608
Enterovirus B	enterovirus B100	EV-B100	BAN2000-10500	DQ902713
Enterovirus B	enterovirus B101	EV-B101	CIV03-10361	AY843308
Enterovirus B	enterovirus B106	EV-B106	148/YN/CHN/12 (China/2012)	KF990476
Enterovirus B	enterovirus B107	EV-B107	TN94-0349	AB426609
Enterovirus B	enterovirus B111	EV-B111	Q0011/XZ/CHN/2000	KF312882
Enterovirus B	enterovirus B112	EV-B112	GAB130 (chimpanzee/Gabon)	KJ418244
Enterovirus B	enterovirus B113	EV-B113	GAB653 (mandrill/Gabon)	KJ701249
Enterovirus B	enterovirus B114; simian agent 5	EV-B114	B165 (vervet monkey)	AF326751
Enterovirus C	poliovirus 1	PV1	Mahoney	V01149
Enterovirus C	poliovirus 1	PV1	Sabin (LSc-2ab)	V01150
Enterovirus C	poliovirus 2	PV2	Lansing (Michigan/37)	M12197
Enterovirus C	poliovirus 2	PV2	Sabin (P712- Ch-2ab)	X00595
Enterovirus C	poliovirus 3	P-3	Leon (California/37)	K01392
Enterovirus C	poliovirus 3	PV3	Sabin (Leon 12a-1-b)	X00925
Enterovirus C	coxsackievirus A1	CVA-1	T.T. (Tompkins) (Coxsackie/NY/47)	AF499635
Enterovirus C	coxsackievirus A11	CVA-11	Belgium 1 (Belgium/51)	AF499636
Enterovirus C	coxsackievirus A13	CVA-13	Flores (Mexico/52)	AF499637
Enterovirus C	coxsackievirus A13; coxsackievirus A18	CVA-13	G-13 (South Africa/50)	AF499640
Enterovirus C	coxsackievirus A17	CVA-17	G12 (South Africa/51	AF499639
Enterovirus C	coxsackievirus A19	CVA-19	NIH-8663 (Dohi) (Japan/52)	AF499641
Enterovirus C	coxsackievirus A20	CVA-20	IH-35 (New York/55)	AF499642

Enterovirus C	coxsackievirus A21	CVA-21	Kuykendall (California/52)	AF546702
Enterovirus C	coxsackievirus A22	CVA-22	Chulman (New York/55)	AF499643
Enterovirus C	coxsackievirus A24	CVA-24	Joseph	EF026081
Enterovirus C	enterovirus C96	EV-C96	BAN00-10499	EF015886
Enterovirus C	enterovirus C99	EV-C99	USA-GA84-10636	EF555644
Enterovirus C	enterovirus C102	EV-C102	BAN99-10424	EF555645
Enterovirus C	enterovirus C105	EV-C105	PER153 (Peru/2010)	JX393302
Enterovirus C	enterovirus C109	EV-C109	NICA08-4327	GQ865517
Enterovirus C	enterovirus C113	EV-C113	BBD-48/Bangladesh/2009	KC344833
Enterovirus C	enterovirus C116	EV-C116	126/Russia/2010	JX514942
Enterovirus C	enterovirus C117	EV-C117	LIT22/Vilnius (Lithuania/2011)	JX262382
Enterovirus C	enterovirus C118	EV-C118	ISR10 (Israel/2011)	JX961708
Enterovirus D	enterovirus D68	EV-D68	Fermon	AY426531
Enterovirus D	enterovirus D70	EV-D70	J670/71 (Japan/71)	D00820
Enterovirus D	enterovirus D94	EV-D94	E210 (Egypt)	DQ916376

Table S6 Phylogenetical classification of the previously tested CVB5 variants[2] by VP1, 5'UTR, non-structural proteins, and 3'UTR regions. The variants were classified into two genogroups A and B according to the bootstrapped and neighbor-joining phylogenetic trees with 1000 replicates constructed by MEGA X software[3]. Highlighted letters indicate that the classification of a certain variant is as same as the VP1-based classification.

**Variant**	**VP1[a]**	**5'UTR**	**Non-structural proteins**	**3'UTR**
CVB5.1	A	A	B	B
CVB5.2	A	A	B	B
CVB5.3	B	B	A	A
CVB5.4	A	A	A	A
CVB5.5	A	A	A	A
CVB5.6	B	B	B	B
CVB5.7	B	B	B	A
CVB5.8	B	B	B	A
CVB5.9	A	A	A	A
CVB5.10	B	B	B	B
CVB5.11	A	A	B	B
CVB5.12	B	B	B	B
CVB5.Faulkner	A	B	A	A

[a] The results of classification is according to our previous studies [2].

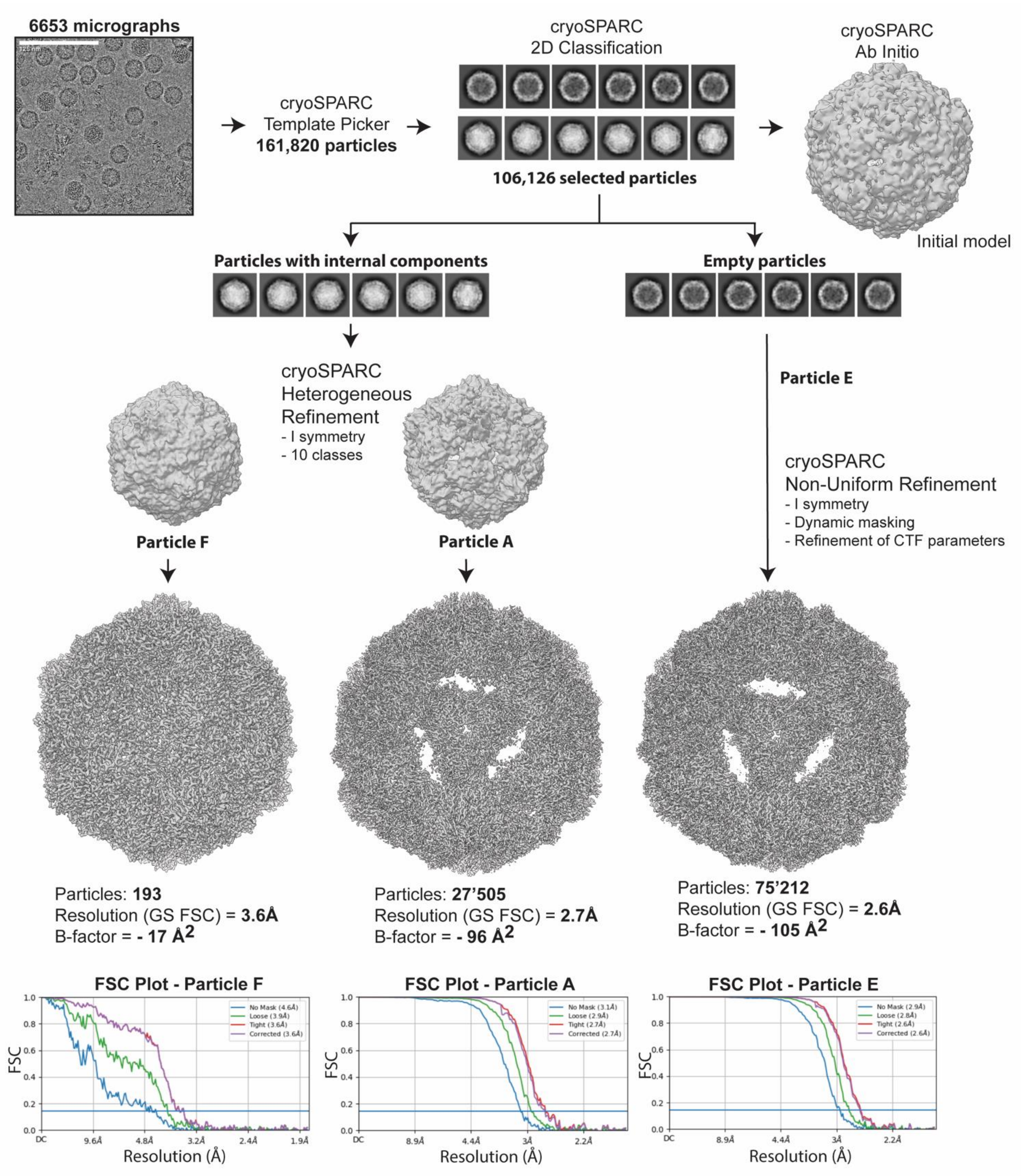

Figure S1 The workflow used to process cryoEM data.

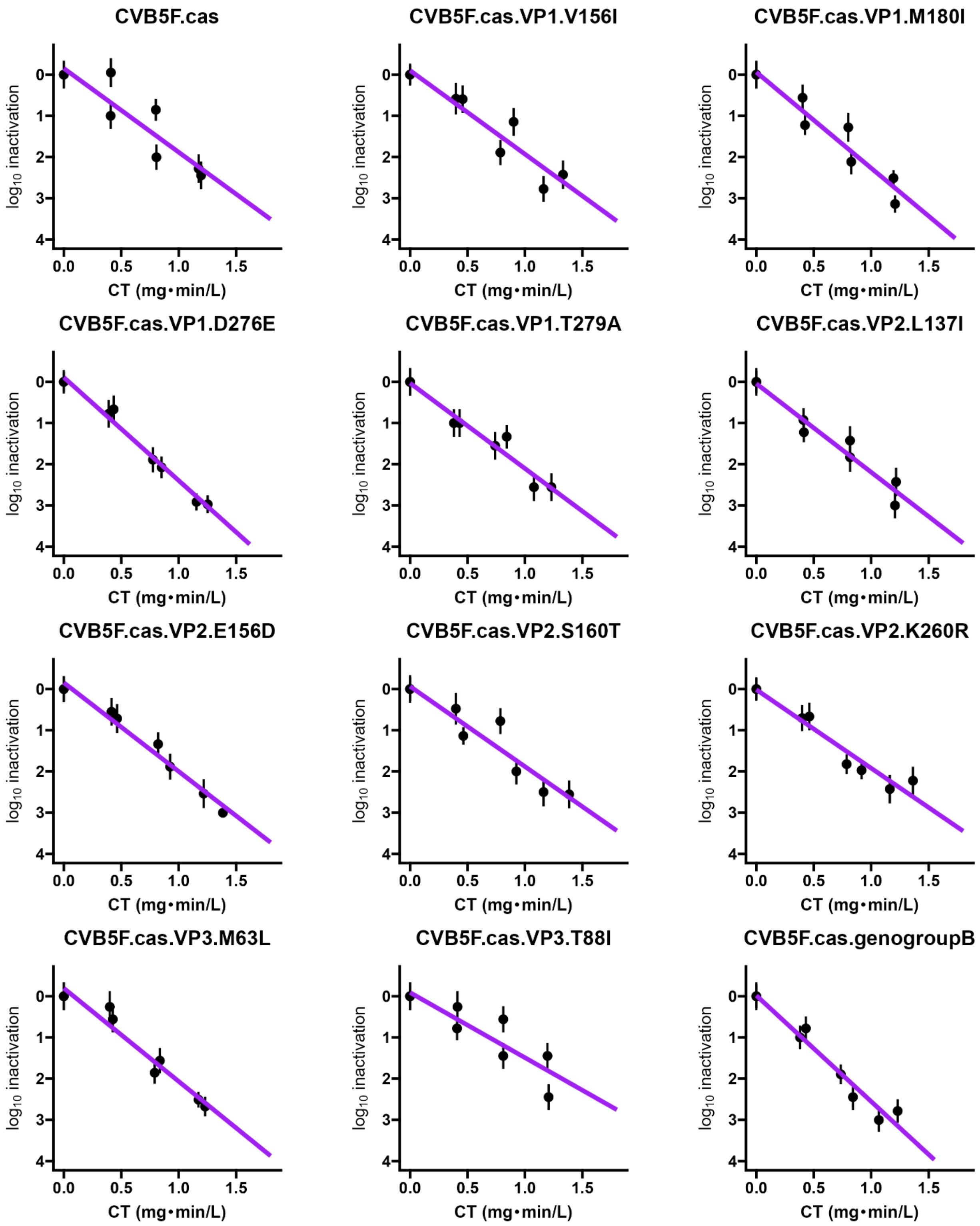

Figure S2 Inactivation curves for free chlorine. The experiments were performed at pH 7.0 at 20°C. The purple line indicates the regression line. The $\log_{10}$ inactivation was plotted versus CT values (mg min L^{-1}) along with error bars representing estimated standard deviation of MPN values as an indicator for measurement uncertainity[4].

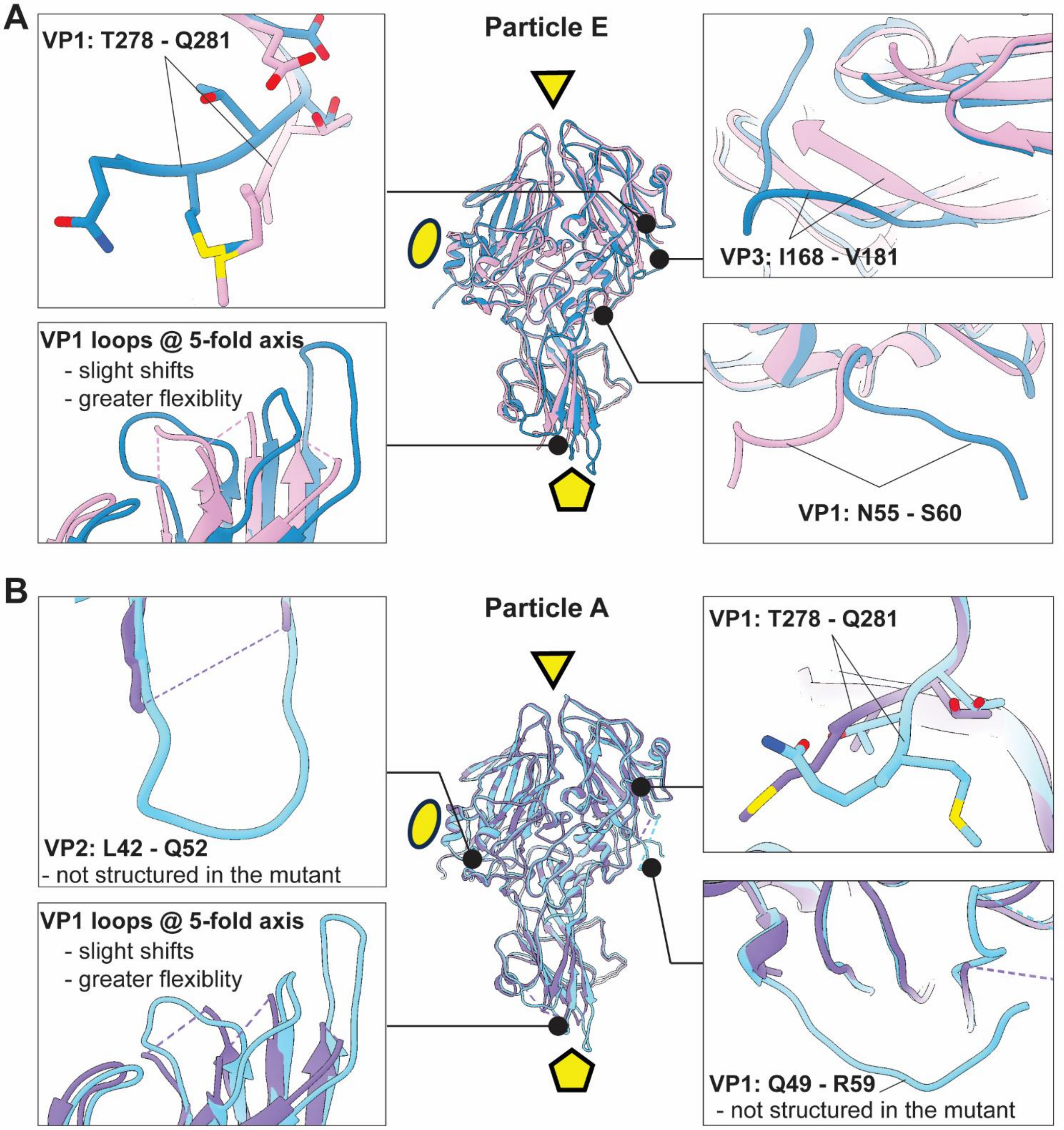

Figure S3 (A) Comparison of the E states in CVB5F.cas.genogroupB (plum) to the previously published structures of the E state CVB5 (PDB IDs: 7WL3; blue). (B) Comparison of the A states in CVB5F.cas.genogroupB (purple) to the previously published structures of the A state CVB5 (PDB IDs: 7XB2; light blue). In both panels, the overlay of the two structures is shown in the middle, and the close-up views of the most notable differences are on each side. For simplicity, only 1 protomer is shown with the locations of 2-, 3-, and 5-fold symmetry axis indicated with ellipse, triangle and pentamer, respectively.

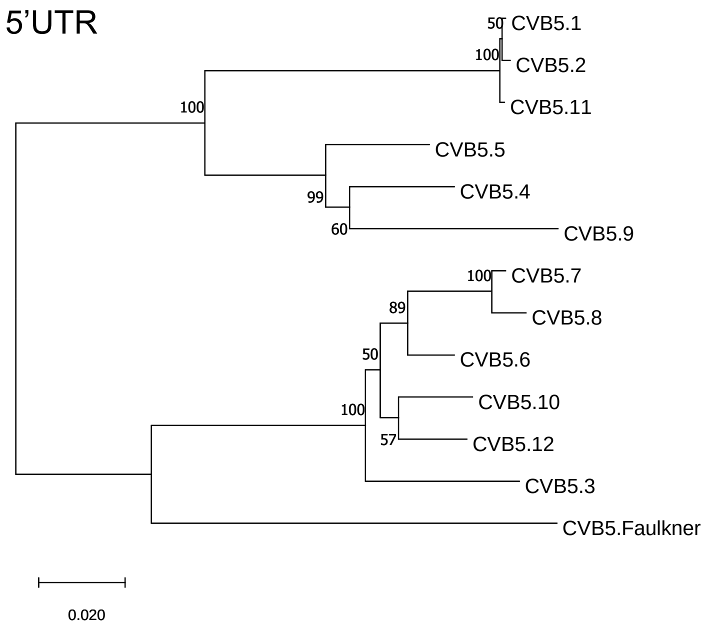

Figure S4 Phylogenetic tree constructed for 5'UTR region of CVB5 variants. The percentages of the replicate trees in which the associated taxa clustered together in the bootstrap test (1000 replicates) are shown next to the branches.

References

1. International Committee on Taxonomy of Viruses: ICTV. *Virus Metadata Resource (VMR): VMR_MSL38_v2.Xlsx*. https://ictv.global/vmr/current (2023).

2. Torii, S. *et al.* Impact of the heterogeneity in free chlorine, UV254, and ozone susceptibilities among coxsackievirus B5 on the prediction of the overall inactivation efficiency. *Environ. Sci. Technol.* **55**, 3156–3164 (2021).

3. Kumar, S., Stecher, G., Li, M., Knyaz, C. & Tamura, K. MEGA X: Molecular evolutionary genetics analysis across computing platforms. *Mol. Biol. Evol.* **35**, 1547–1549 (2018).

4. Jarvis, B., Wilrich, C. & Wilrich, P. T. Reconsideration of the derivation of Most Probable Numbers, their standard deviations, confidence bounds and rarity values. *J. Appl. Microbiol.* **109**, 1660–1667 (2010).
